# Eight-Electron Reduction of CO2 to CH4 by a Bioinspired Mo-Fe-S Cubane Catalyst

**DOI:** 10.21203/rs.3.rs-11177343/v1

**Published:** 2026-10-14

**Authors:** Kazuki Tanifuji, Yuto Matsuoka, Kodai Yamamoto, Hitoshi Izu, Pabasara Ediriweera, Yilin Hu, Markus Ribbe, W. M. C. Sameera, Yasuhiro Ohki

## Abstract

Metal-sulfur (M-S) clusters are ancient biological cofactors that catalyze CO2 and N2 reduction, yet no biological or synthetic M-S cluster has achieved sustained, high-turnover conversion of CO2 to hydrocarbons at a single active site. We reasoned that effective CO2-to-CH4 catalysis requires not only CO2 activation but also retention of a metal-bound CO intermediate for subsequent six-electron reduction. Here, a bioinspired [Cp*3Mo3S4Fe] cubane (Cp* = C5Me5) catalyzes the direct eight-electron reduction of CO2 to CH4 under ambient conditions with turnover numbers exceeding 150,000 and only trace CO formation. A structurally characterized CO2-derived anionic Fe-CO intermediate was identified, and a neutral Fe-CO cubane undergoes nearly quantitative conversion to CH4 under the catalytic conditions. These results identify synthetic M-S clusters as a competent framework for advancing catalytic CO2 reduction beyond the two-electron stage, with appropriate molecular design and reaction conditions. More broadly, they establish productive retention of a metal-bound CO intermediate as a key molecular design principle for CO2-to-hydrocarbon catalysis.

**Main Text:**

Metal-sulfur (M-S) clusters are among the most ancient cofactors known to biology, with phylogenomic evidence linking them to the reductive activation of $CO_2$ and $N_2$ in the last universal common ancestor of cellular life [1,2]. This reductive chemistry persists in extant enzymes. Carbon monoxide dehydrogenase (CODH) catalyzes the reversible interconversion of $CO_2$ and CO at a Ni-Fe-S active site [3,4,5], while nitrogenases reduce $N_2$ at intricate M-Fe-S cofactors (M = Mo, V, Fe) [6,7,8] and, in the case of vanadium-dependent nitrogenase, further reduce CO to $CH_4$, $C_2H_4$, and short-chain hydrocarbons [9]. Together, these enzymes demonstrate that biological M-S clusters can catalyze both $CO_2$ activation and CO reduction. However, these functions remain largely separated among different enzyme systems. Although some nitrogenases can reduce $CO_2$ directly to hydrocarbons, their turnover numbers (TONs) are below 100 under specialized conditions [10,11,12]. Neither biological nor synthetic M-S clusters have achieved sustained, high-turnover catalytic conversion of $CO_2$ directly to hydrocarbons at a single active site (Fig. 1).

(**Fig. 1**, here)

Synthetic M-S clusters have long been developed as structural and functional models of enzymatic cofactors, yet molecular M-S catalysts remain largely limited to the two-electron reduction of $CO_2$ to CO or formate (Eq. 1) [13,14,15]. Direct conversion of $CO_2$ to $CH_4$ requires six additional electrons and protons to be delivered before the CO intermediate dissociates (Eq. 2). An effective molecular catalyst for this eight-electron transformation must combine a durable cluster framework with an accessible metal site that binds and activates inert small molecules under mild conditions, while retaining the CO-derived intermediate long enough for its further reduction. Our recently developed Mo-Fe-S Cubane [16,17], in which the Fe site catalytically reduces $N_2$ under ambient conditions [18] and exhibits strong Fe-CO interaction [19], provides a molecular platform for testing this design principle.

$$CO_2 + 2e^- + 2H^+ \rightarrow CO + H_2O \qquad \text{(Eq. 1)}$$

$$CO + 6e^- + 6H^+ \rightarrow CH_4 + H_2O \qquad \text{(Eq. 2)}$$

Here we show that a Mo-Fe-S cubane cluster catalyzes the direct eight-electron reduction of $CO_2$ to $CH_4$ under ambient conditions, with TONs exceeding 150,000 and only trace CO formation (Fig. 1). This activity surpasses previous M-S cluster catalysts by approximately four orders of magnitude [20,21,22] and, to our knowledge, represents the highest turnover number reported to date for a homogeneous molecular catalyst for $CO_2$-to-$CH_4$ conversion. Mechanistic studies identify a persistent yet catalytically competent Fe-CO intermediate that undergoes nearly quantitative conversion to $CH_4$ under the catalytic conditions. These findings demonstrate that a single molecular M-S cluster can integrate $CO_2$ activation and complete reduction of the resulting CO intermediate, a chemistry that appears not to have been achieved at a single active site over nearly four billion years of biological evolution. The ability to retain a metal-bound CO intermediate without arresting its further reduction offers a design principle for molecular $CO_2$-to-hydrocarbon catalysis.

We began by evaluating the catalytic performance of the chloride ($Cl^-$)-bound clusters [$Cp^R{}_3Mo_3S_4FeCl$] ($Cp^R$ = Cp* (**1**), $Cp^L$ (**2**), $Cp^{XL}$ (**3**)) [16,17] for $CO_2$ reduction. Reactions were conducted in THF using $SmI_2$ as a single-electron reductant and $H_2O$ as a proton source, and the conditions were selected on the basis of our previous studies of multi-electron small-molecule reduction [20-22] and related Mo-catalyzed $N_2$ reduction to ammonia [23]. Under 1 atm of $CO_2$, the catalytic activity depended strongly on the steric profile of the $Cp^R$ ligands. Among the available $Cp^R$ ligands, the least sterically hindered cluster, [$Cp^*{}_3Mo_3S_4FeCl$] (**1**), showed the highest activity. In the presence of 5,000 equiv. of $SmI_2$ and 1.5% v/v $H_2O$, **1** produced 216 ± 29 equiv. of $CH_4$, corresponding to 35 ± 5% of the consumed reducing equivalents (Table 1, entry 1 and Supplementary Fig. 2) [24]. $H_2$ was formed as the main competing product (1,729 ± 60 equiv., 69 ± 10% selectivity), whereas CO formation was negligible (3.4 ± 0.9 equiv., 0.54 ± 0.14% selectivity). By contrast, the more sterically demanding clusters **2** ($Cp^L$) and **3** ($Cp^{XL}$) produced only 10 ± 1.1 and 0.4 ± 0.2 equiv. of $CH_4$ (Table 1, entries 2 and 3). In these reactions, 87-88% of the reducing equivalents were used for $H_2$ formation (2,170 ± 40 and 2,190 ± 140 equiv., respectively). These results identified **1** as the most effective catalyst among the three cluster variants and suggested that steric accessibility around the Fe site of the cubane is critical for $CO_2$ reduction.

(**Table 1**, here)

We next examined whether the observed activity could arise from iron salts, cluster fragments, or decomposition products. The individual synthetic precursors of **1**, $[Cp^*{}_3Mo_3S_4]$ and $FeCl_2$, each produced less than 2.6 equiv. of $CH_4$ under otherwise identical conditions (Table 1, entries 4 and 8). Common mononuclear and dinuclear iron complexes, including Fe(TPP)Cl (TPP = *meso*-tetraphenylporphyrin), $Fe_2(CO)_9$, and $FeCp_2$ (Cp = $C_5H_5$), were also inactive or weakly active. $FeCp_2$ gave at most 7 equiv. of $CH_4$ (Table 1, entries 5-7). Fe(TPP)Cl, despite its established activity in electro- and photocatalytic $CO_2$-to-CO conversion [25,26], did not produce appreciable CO or $CH_4$ under the present conditions (Table 1, entry 5). These control experiments indicate that neither simple iron species nor the separated components of the cubane account for the catalytic activity of **1**.

Isotope labelling experiments using $^{13}CO_2$ established the carbon source of the $CH_4$ product. Gas chromatography-mass spectrometry (GC-MS) analysis showed identical retention times for $CH_4$ generated from $^{12}CO_2$ and $^{13}CO_2$, but the molecular-ion signal shifted from $m/z$ = 16 for $^{12}CH_4$ to $m/z$ = 17 for $^{13}CH_4$ (Supplementary Fig. 3). The corresponding +1 $m/z$ shift was also observed throughout the fragmentation pattern (Fig. 2A). These results establish that the carbon atom of $CH_4$ originates from $CO_2$.

(**Fig. 2**, here)

Time-course measurements under the standard conditions showed that $CH_4$ and $H_2$ were formed linearly during the initial 10 min, with initial turnover frequencies (TOFs) of 10 $min^{-1}$ and 148 $min^{-1}$, respectively (Fig. 2B and Supplementary Fig. 4). Product formation approached a plateau after approximately 30 min, by which point 97% of the $SmI_2$ reductant had been consumed. The termination of product formation coincided with depletion of the electron source ($SmI_2$), rather than with an observable loss of catalytic activity. This conclusion is further supported by the large-scale experiments discussed below, which demonstrate sustained activity in the presence of a large excess reductant. Polynuclear clusters are often prone to degradation and aggregation, but our kinetic data support the molecular nature of the catalysis. The initial rates of both $CH_4$ and $H_2$ formation were proportional to the concentration of **1** over the range of 5 - 40 μM, establishing a first-order dependence on the cluster concentration over this range (Fig. 2C and Supplementary Fig. 5). Furthermore, no induction period was observed. Although these kinetic features alone do not exclude every possible decomposition pathway, they are compatible with catalysis by a molecular species derived directly from **1** rather than by slowly formed nanoparticles or other aggregated species.

Additional evidence for persistence of the $[Cp^*{}_3Mo_3S_4Fe]$ cubane core came from post-catalytic mass spectrometry (MS) analysis. The MS spectrum of the reaction mixture after $CO_2$

reduction displayed four major mono-cationic signals (i-iv in Supplementary Fig. 7). Signal (i), with its most intense peak at $m/z$ = 1003.7, matched the calculated isotope pattern of $[Cp^*{}_3Mo_3S_4FeI]^+$, and no prominent signals attributable to decomposition products of the $[Cp^*{}_3Mo_3S_4Fe]$ core were detected. Signals (ii)-(iv) were assigned on the basis of their isotope patterns to oxidized and oligomerized $SmI_2$-derived species, $[Sm_2I_5(OH)(THF)_3]^+$ (ii), $[Sm_2I_5(OH)(THF)_3(H_2O)_3]^+$ (iii), and $[Sm_3I_8(OH)(THF)_3(H_2O)_3]^+$ (iv). Related oligomeric products of oxidized $SmI_2$ have been structurally characterized previously [23]. Overall, these results support retention of the $[Cp^*{}_3Mo_3S_4Fe]$ core during catalysis.

Attempts to observe catalytic intermediates directly under turnover conditions were unsuccessful, consistent with their low steady-state concentrations. We therefore sought to intercept intermediates under stoichiometric conditions. When cluster **1** was reduced with 10 equiv. of $KC_8$ under a $CO_2$ atmosphere (1 atm), the anionic CO-adduct $[Cp^*{}_3Mo_3S_4Fe(CO)]^-$ (**4**) was formed in 32% NMR yield (Fig. 2D and Supplementary Fig. 8). This reaction was carried out under anhydrous conditions to arrest the reduction at an intermediate stage. The absence of a proton source, which would otherwise promote further reduction of the Fe-bound CO ligand, likely contributed to the accumulation of **4**. X-ray crystallographic analysis established **4** as the potassium salt of an anionic Fe-CO cubane (Fig. 2E and Supplementary Fig. 23). The CO stretching band of **4** appeared at 1690-1692 $cm^{-1}$ in the infrared (IR) spectra (Supplementary Figs. 9 and 17). Preparation of **4** under $^{13}CO_2$ (Supplementary Fig. 10) shifted this band to 1648 $cm^{-1}$ ($\Delta_\nu$ = 42-44 $cm^{-1}$, Fig. 2F and Supplementary Fig. 11), in agreement with the expected isotopic shift for a $^{13}C$-labeled CO ligand ($\Delta_\nu$ = 37 $cm^{-1}$). These results demonstrate that the Fe-bound CO ligand is derived from $CO_2$ and support the involvement of an Fe-CO intermediate in the reduction pathway.

The catalytic relevance of the Fe-CO intermediate was established independently. Both anionic cluster **4** and neutral CO cluster $[Cp^*{}_3Mo_3S_4Fe(CO)]$ (**5**) catalyzed $CO_2$ reduction under the standard conditions, producing 273 ± 34 and 281 ± 41 equiv. of $CH_4$, respectively (Table 1, entries 9 and 10). These activities were comparable to or greater than that of chloride precursor **1**. Cyclic voltammetry of **5** revealed a reversible redox couple assignable to the **5**/**4** couple at –1.73 V vs. $Fc/Fc^+$ (Fc = $FeCp_2$) (Supplementary Fig. 18), corresponding to –1.16 V vs. SCE (SCE = saturated calomel electrode) (Supplementary Fig. 19) [27,28]. This potential is accessible by the $SmI_2/H_2O$ reducing system (–1.3 V vs. SCE) [29], suggesting that cluster **5** is reduced to **4** under catalytic conditions. Characterization data for independently synthesized **4** and **5** are summarized in Supplementary Figs. 12- 17, 23, 24, and Supplementary Table 4.

Most importantly, stoichiometric treatment of independently synthesized **5** with excess $SmI_2$ (32 equiv.) and protons ($H_2O$) under argon converted its Fe-bound CO into $CH_4$ in near-quantitative yield (0.96 ± 0.08 equiv.; Fig. 2G and Supplementary Table 2). The Fe-CO species is therefore not an off-cycle decomposition product or terminal resting state, but a chemically and catalytically competent intermediate in $CH_4$ formation.

The Fe-CO unit is persistent in the absence of a proton source. The CO ligand of neutral Fe-CO cluster **5** was not detectably displaced after 1 day at room temperature under $CO_2$, as monitored by $^1H$ NMR in THF-$d_8$ (Supplementary Fig. 20). Its CO stretching frequency ($\nu_{CO}$) of 1857 $cm^{-1}$ is substantially lower than that of free CO (2143 $cm^{-1}$), whereas the anionic Fe-CO cluster **4** exhibits an even lower value of 1690-1692 $cm^{-1}$ (Supplementary Figs. 9, 14, and 17). The $\nu_{CO}$ values of both **4** and **5** rank among the lowest reported for Fe-CO species bearing cubic metal-sulfur cores (Supplementary Fig. 21) [19,30,31,32,33,34]. Moreover, the $\nu_{CO}$ of **4** lies at the lowest end of structurally characterized terminal Fe-CO complexes, below even that of $[Fe(CO)_4]^{2-}$ (1730 $cm^{-1}$, Supplementary Fig. 22) [35,36]. The unusually low $\nu_{CO}$ values of **4** point to strong Fe→CO $\pi$ backdonation and substantial activation of the bound CO ligand. While strong metal-CO interactions can lead to thermodynamic trapping or catalyst inhibition, the

[$Cp^*{}_3Mo_3S_4Fe$] framework appears to enable productive retention, maintaining the activated Fe-CO intermediate for subsequent reduction toward $CH_4$ rather than simply promoting CO dissociation. This behavior provides an interesting contrast with CODH, where CO is released as product, and nitrogenase, where CO can inhibit catalysis, highlighting how the surrounding metal-sulfur framework can tune the balance among CO binding, activation, and further reduction. This productive retention, rather than the strength of CO binding alone, accounts for the low yield of CO and its continued reduction to $CH_4$.

Based on the experimental observations and density functional theory (DFT) calculations, we propose the catalytic cycle illustrated in Fig. 3. Consistent with the observed first-order kinetics (Fig. 2C and Supplementary Fig. 5), the catalysis proceeds via a unimolecular pathway. Reduction of chloride precursor **1** induces loss of $Cl^-$, generating a coordinatively unsaturated Fe site capable of binding $CO_2$. Theoretical calculations support thermodynamically favorable binding of $CO_2$ to the vacant site in a side-on ($\eta^2$) fashion (see details in the Supplementary Information and Supplementary Figs. 25 and 26). The computed $CO_2$ binding energy is 31.4 kcal/mol for [$Cp^*{}_3Mo_3S_4Fe(CO_2)$], relatively higher than the value calculated for the $Cp^{XL}$ analog (21.4 kcal/mol). Bulkier ligands on the Mo atoms thus yield smaller $CO_2$ binding energy, providing a rationale for markedly lower $CO_2$ reduction activities of clusters **2** ($Cp^R = Cp^L$) and **3** ($Cp^R = Cp^{XL}$). Subsequent transfer of two electrons and two protons promotes C-O bond cleavage and $H_2O$ release to form the neutral CO cluster **5**, which then undergoes one-electron reduction to give the corresponding anionic cluster **4**. The preferential binding of CO over $CO_2$ at **4** and **5** is also bolstered by DFT-calculated binding energies of CO (73.7 and 55.8 kcal/mol for **4** and **5**, respectively, vs. 32.3 kcal/mol for [$Cp^*{}_3Mo_3S_4Fe(CO_2)$]) (see details in the Supplementary Information). Further proton- and electron-transfer steps convert the bound CO ligand into $CH_4$ via an Fe-$CH_3$ intermediate, regenerating the active species. The regenerated coordinatively unsaturated Fe site should also be competent for proton reduction. Under argon, cluster **1** produced $H_2$ with an initial TOF of 1,116 $min^{-1}$, whereas under $CO_2$ this value decreased to 148 $min^{-1}$ (Supplementary Fig. 4). In contrast, the sterically congested cluster **3** ($Cp^{XL}$) exhibited an $H_2$-formation TOF exceeding 2,000 $min^{-1}$ even under $CO_2$ (Supplementary Fig. 6). These observations are in line with competition between $CO_2$ binding and proton reduction within the same molecular catalyst. Access of $CO_2$ to the cubane suppresses, but does not eliminate, the competing $H_2$ evolution pathway. This competition parallels the intrinsic proton-reduction chemistry of nitrogenase, which obligatorily evolves $H_2$ during catalysis and relies on ATP-coupled electron transfer to drive difficult multielectron substrate reductions. In this context, achieving the eight-electron reduction of $CO_2$ to $CH_4$ with up to 35% selectivity under ambient conditions, without ATP-dependent protein machinery, underscores the remarkable intrinsic catalytic capability of the isolated [$Cp^*{}_3Mo_3S_4Fe$] framework. This performance does not arise from a single structural or electronic property. Rather, it emerges from the interplay of an accessible reaction site, a durable metal-sulfur framework, efficient binding and activation of inert small molecules under mild conditions, and productive retention of the CO-derived intermediate.

(**Fig. 3**, here)

Finally, to demonstrate the exceptional durability of the [$Cp^*{}_3Mo_3S_4Fe$] framework, we scaled up the catalytic reaction using a massive excess of reductant (4 million equiv. of $SmI_2$) with chloride precursor **1** and neutral Fe-CO cluster **5**. Optimization studies revealed that the reaction temperature strongly influenced $CH_4$ production. At room temperature, cluster **1** produced only 19,000 ± 2,100 equiv. of $CH_4$ (Supplementary Table 3, entry 1). At 0 °C, however, cluster **1** produced 127,000 ± 5,000 equiv. of $CH_4$, whereas neutral Fe-CO cluster **5** reached 157,000 ± 5,000 equiv. with no apparent catalyst deactivation before depletion of the available reducing equivalents (Table 1, entries 11 and 12 and Supplementary Table 3, entries 2 and 3). These turnover numbers exceed those of previously reported M-S cluster catalysts for hydrocarbon formation by approximately four orders of magnitude. Our earlier [$Fe_4S_4$]-based systems, for example, were limited to fewer than 25 turnovers under related chemically reducing conditions [20-22]. More broadly, homogeneous molecular catalysts for $CO_2$ reduction predominantly produce the two-electron reduction products, such as CO and formate [37,38], and molecular $CO_2$-to-$CH_4$ conversion catalysis remains uncommon. Among the reported systems, a nickel macrocyclic complex achieved 29,000 turnovers of $CH_4$ over 72 hours, although CO (130,000 turnovers) was the dominant product under photochemical conditions [39]. A trimethylammonium-substituted iron tetraphenylporphyrin complex afforded 79 turnovers of $CH_4$ together with 367 turnovers of CO under photocatalytic conditions [40,41]. Direct comparison between chemical and photochemical systems requires caution because reductants, energy inputs, and efficiency metrics differ. Nevertheless, the combination of more than 150,000 turnovers and only trace CO formation distinguishes the present Mo-Fe-S cubane from previously reported homogeneous molecular catalysts.

In conclusion, a bioinspired [$Cp^*{}_3Mo_3S_4Fe$] cubane catalyzes the direct eight-electron reduction of $CO_2$ to $CH_4$ under ambient pressure with turnover numbers exceeding 150,000. A single molecular metal-sulfur cluster can mediate both $CO_2$-to-CO conversion and the subsequent six-electron reduction of the resulting Fe-bound CO intermediate with exceptional durability. A structurally characterized $CO_2$-derived anionic Fe-CO intermediate and the near-quantitative conversion of an independently synthesized neutral Fe-CO cluster to $CH_4$ collectively establish the catalytic competence of the Fe-CO intermediate. These findings identify productive retention of a metal-bound CO intermediate, coupled with its continued reduction rather than CO release, as an important molecular design principle for $CO_2$-to-hydrocarbon catalysis. Moreover, unlike metalloenzymes, which use structured hydrogen-bonding networks and proton relays to control proton delivery, the [$Cp^*{}_3Mo_3S_4Fe$] system sustains effective multi-electron/multi-proton reduction using only $H_2O$ in THF. This behavior highlights the intrinsic ability of the metal-sulfur framework to coordinate proton and electron transfer without a protein scaffold. Future incorporation of pendant proton relays or other secondary-sphere features could provide additional control over proton delivery and potentially suppress competing $H_2$ evolution.

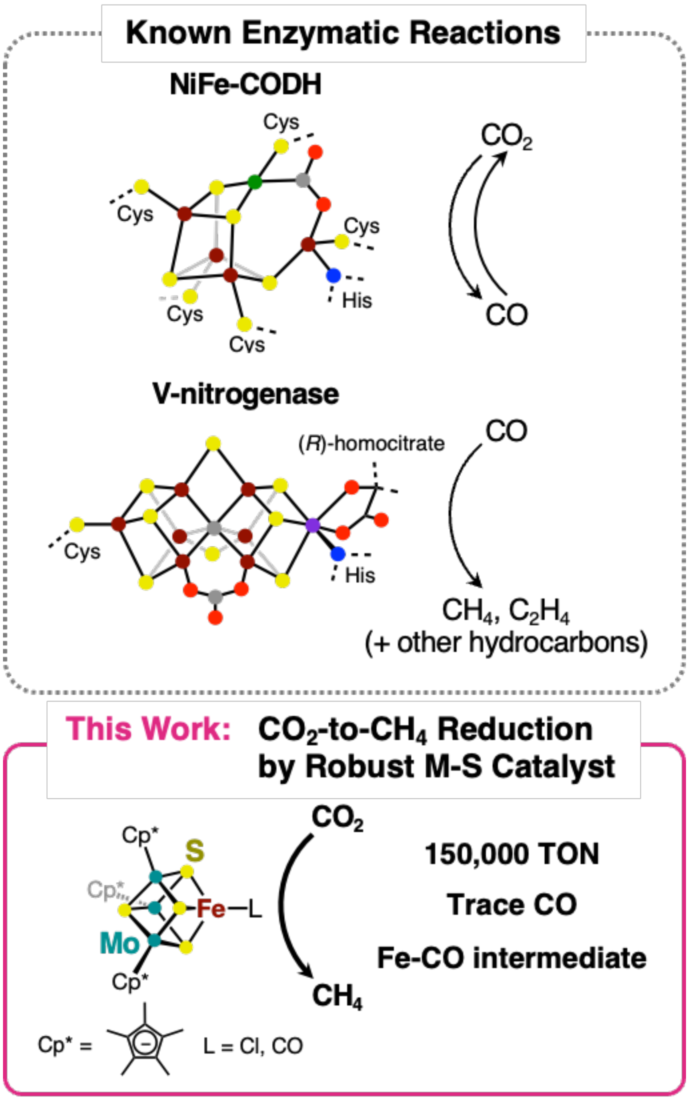

**Fig. 1. $CO_2$ reduction to $CH_4$ catalyzed by synthetic metal-sulfur clusters with comparison to enzymatic reactions.** (Top) NiFe-carbon monoxide dehydrogenase (NiFe-CODH) catalyzes the interconversion of $CO_2$ and CO, while vanadium-dependent nitrogenase (V-nitrogenase) reduces CO to $CH_4$, $C_2H_4$, and other hydrocarbons. The metal-sulfur active sites are shown with atoms colored as follows: Fe, reddish brown; Mo, teal; Ni, green; V, purple; S, yellow: O, red; C, gray; N, blue. (Bottom) The cubane-type Mo-Fe-S clusters, [$Cp^*_3Mo_3S_4Fe(L)$] ($Cp^*$ = $C_5Me_5$, L = Cl, CO), catalyze $CO_2$-to-$CH_4$ reduction with exceptional turnover numbers (TONs) and trace CO formation. Stoichiometric reactions and catalytic evaluations identified Fe-CO species as intermediates.

**Table 1. Catalytic reduction of $CO_2$ to $CH_4$ using [$Cp^*_3Mo_3S_4Fe$] clusters and comparison of catalytic performance with relevant cluster and iron compounds.** Reaction conditions: (entries 1-10) precatalyst (10 nmol), $SmI_2$ (50 μmol), $H_2O$ (1.5% v/v); (entries 11 and 12) precatalyst (0.040 nmol), $SmI_2$ (0.16 mmol), $H_2O$ (6.25% v/v).

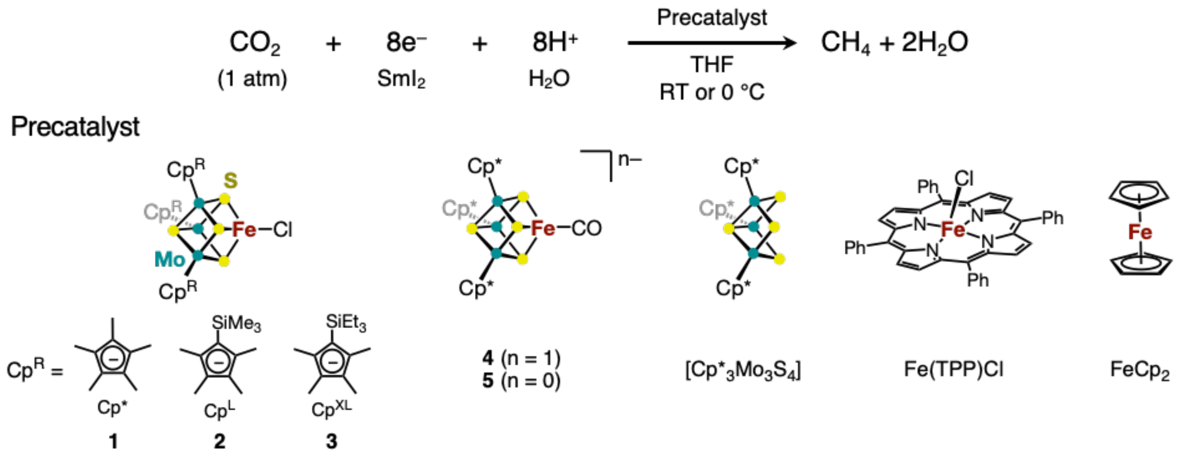

| Entry | Precatalyst | $SmI_2$ (equiv.)[a] | Product (equiv.)[a] [Selectivity (%)][b] | | |
|---|---|---|---|---|---|
| | | | $CH_4$ | CO | $H_2$ |
| 1[c] | **1** | 5000 | 216 ± 29 [35] | 3.4 ± 0.9 [0.54] | $1.72 \cdot 10^3$ ± 60 [69] |
| 2[c] | **2** | 5000 | 10 ± 1.1 [1.6] | n.d. | $2.17 \cdot 10^3$ ± 40 [87] |
| 3[c] | **3** | 5000 | 0.4 ± 0.2 [0.06] | n.d. | $2.19 \cdot 10^3$ ± 140 [88] |
| 4 | [$Cp^*_3Mo_3S_4$] | 5000 | n.d. | n.d. | $2.17 \cdot 10^3$ [87] |
| 5 | Fe(TPP)Cl | 5000 | n.d. | 4.9 [0.20] | $1.60 \cdot 10^3$ [64] |
| 6 | $FeCp_2$ | 5000 | 7.0 | 2.1 [0.08] | $1.38 \cdot 10^3$ [55] |
| 7 | $Fe_2(CO)_9$ | 5000 | 5.9 | 6.3 [0.25] | $1.96 \cdot 10^3$ [78] |
| 8 | $FeCl_2$ | 5000 | 2.6 | n.d. | $1.94 \cdot 10^3$ [78] |
| 9[c] | **4** | 5000 | 281 ± 41 | 3.7 ± 1.2 [0.15] | $1.17 \cdot 10^3 \pm 1.6 \cdot 10^2$ [47] |
| 10[c] | **5** | 5000 | 272 ± 35 | 5.7 ± 3.4 [0.23] | $1.25 \cdot 10^3 \pm 1.1 \cdot 10^2$ [50] |
| 11[c,d] | **1** | $4.0 \cdot 10^6$ | $1.27 \cdot 10^5 \pm 5 \cdot 10^3$ [25] | $9.3 \cdot 10^2 \pm 3.6 \cdot 10^2$ [0.05] | $1.18 \cdot 10^6 \pm 1.4 \cdot 10^5$ [59] |
| 12[c,d] | **5** | $4.0 \cdot 10^6$ | $1.57 \cdot 10^5 \pm 5 \cdot 10^3$ [31] | $1.8 \cdot 10^3 \pm 2 \cdot 10^2$ [0.09] | $1.09 \cdot 10^6 \pm 6 \cdot 10^4$ [55] |

[a] equiv. per precatalyst. [b] $SmI_2$-based selectivity. Selectivity was calculated on an electron-balance basis, requiring 8, 2, and 2 electrons per molecule of $CH_4$, CO, and $H_2$. Values are shown in brackets. [c] Data were obtained from three individual experiments and are presented as average ± standard deviation. [d] Reactions were performed at 0 °C.

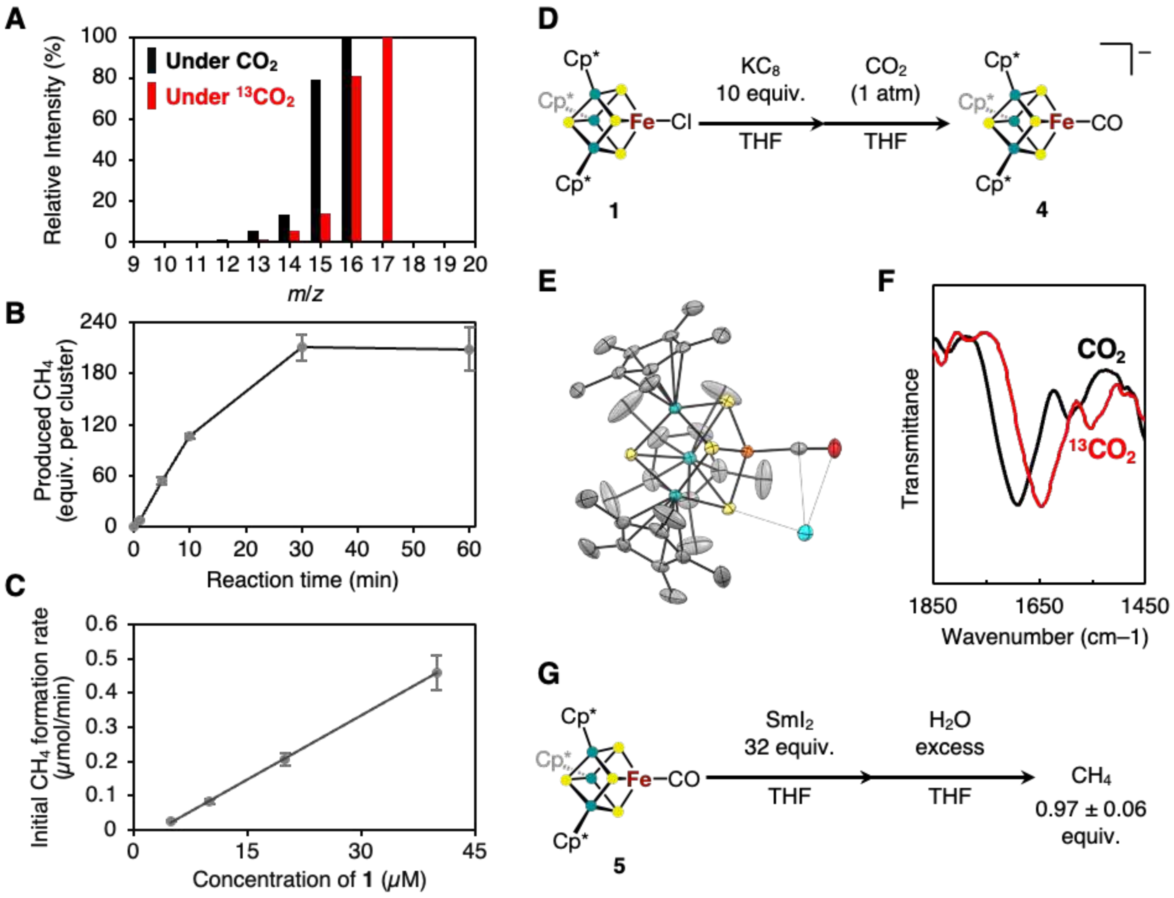

**Fig. 2. Isotope labeling and kinetic characterization of the catalytic $CO_2$ reduction by 1 (A-C), isolation and characterization of a CO-bound intermediate (D-F), and stoichiometric conversion of bound CO to $CH_4$ (G).** (**A**) Mass spectra of $CH_4$ produced under unlabeled $CO_2$ (black) and $^{13}CO_2$ (red), analyzed by gas chromatograph-mass spectrometry (GC-MS). The +1 *m*/*z* shift under $^{13}CO_2$ confirms that the carbon of $CH_4$ originates from $CO_2$. (**B**) Time course of $CH_4$ formation during the reaction of **1** with $SmI_2$ and $H_2O$ under $CO_2$. Product accumulation plateaus at approximately 30 min, coinciding with consumption of the $SmI_2$ reductant. (**C**) Initial rate of $CH_4$ formation as a function of the concentration of **1** (5-40 μM). The linear dependence ($R^2$ = 0.9997) indicates first-order kinetics with respect to cluster **1**. (**D**) Formation of CO-bound cluster **4** upon treatment of **1** with $KC_8$ under $CO_2$ atmosphere. (**E**) Crystal structure of **4** shown with thermal ellipsoids at 50% probability. Hydrogen atoms are omitted for clarity. Shown is one of the two crystallographically independent pairs of **4** with a potassium cation in the asymmetric unit (For more details, see Supplementary Fig. 23). Color legend: C, grey; O, red; K, turquoise; S, yellow; Mo, teal; and Fe, orange. (**F**) Solid-state IR spectra of **4** in the CO-stretching region, prepared under $CO_2$ (black) and $^{13}CO_2$ (red) atmospheres. (**G**) Quantitative methane formation from CO-bound cluster **5** upon reaction with $SmI_2$ and $H_2O$ under an Ar atmosphere.

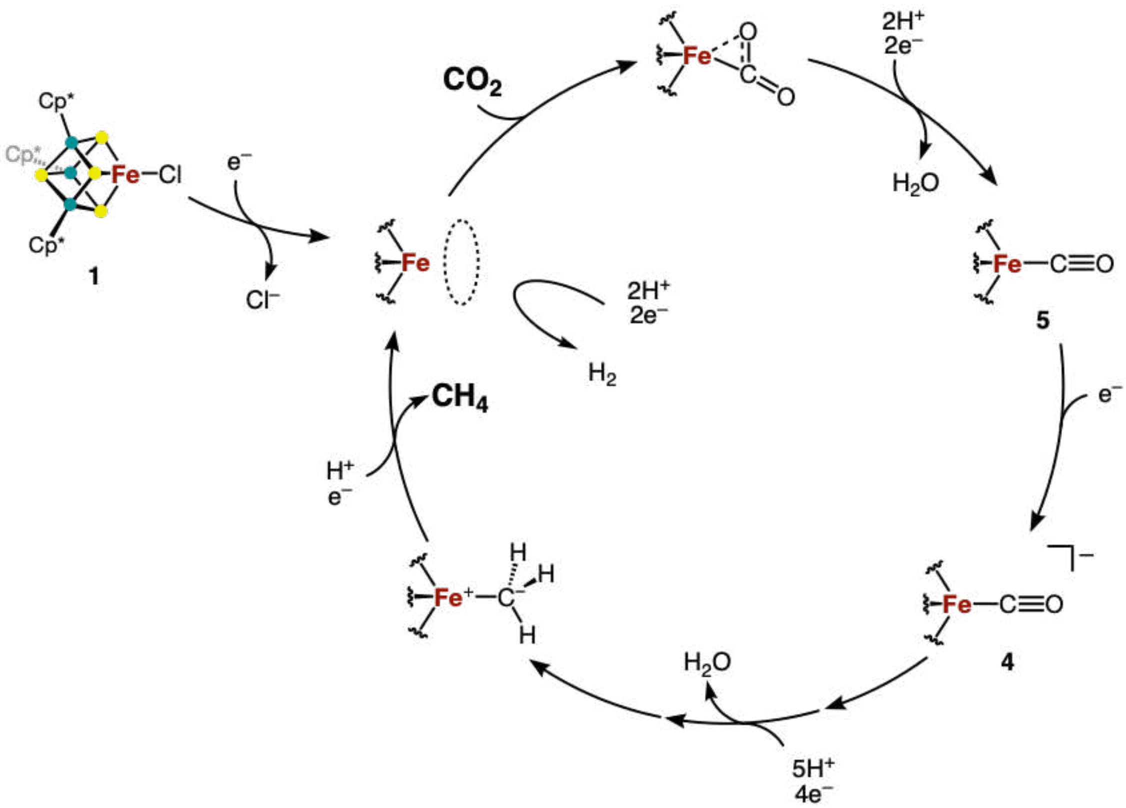

**Fig. 3. Proposed catalytic cycle of $CO_2$-to-$CH_4$ conversion by the [Cp*$_3$Mo$_3$S$_4$Fe] cluster.** Reduction of Cl-bound precatalyst **1** releases $Cl^-$ and generates a coordinatively unsaturated Fe site. This site binds $CO_2$, which is reduced to $CH_4$ through Fe-CO intermediates **5** and **4** by successive electron and proton transfer. Release of $CH_4$ regenerates the vacant Fe site to close the cycle. The unsaturated Fe site also mediates $H^+$ reduction to $H_2$ as a competing pathway.

**Acknowledgments:**

We thank S. Muramatsu (Hiroshima University) for attempted mass spectrometry measurements of **4** and **5**. We also acknowledge supercomputing resources of the Institute for Chemical Research at Kyoto University.

**Funding:** This work was financially supported by Grant-in-Aids for Scientific Research (Grant No. 22K05143 for K.T., 23K13763 and 25K18050 for H.I., 25K22274 and 26K21755 for Y.O.), Grant-in-Aids for JSPS Fellows (Grant No. 24KJ1479 for Y. M.), Transformative Research Areas (A) JP24A202 Integrated Science of Synthesis by Chemical Structure Reprogramming (Grant No. 25H02027 for K.T.) from the Japanese Ministry of Education, Culture, Sports, Science and Technology (MEXT), and CREST grant (Grant No. JPMJCR21B1 for Y.O.) from Japan Science and Technology Agency (JST). M.W.R. and Y.H. were supported by NIH-NIGMS R35 grant GM161209 (to M.W.R.) and NIH-NIGMS R35 grant GM161208 (to Y.H.). We are also grateful for financial support from Toshiaki Ogasawara Memorial Foundation (for Y.O.), the Takeda Science Foundation, Toyota Riken Scholar Program, and "Innovation inspired by Nature" Research Support Program (SEKISUI CHEMICAL CO., LTD) (for K.T.), International Collaborative Research Program of ICR, Kyoto University (for Y.O. and W. M. C. S. and for K. T., M. W. R., and Y. H.).

**Authors contributions:** Conceptualization: K. T., Y. O.; Experimental data acquisition: Y.M., K.Y., H.I.; DFT calculations: P.E., W.M.C.S.; Funding acquisition: Y.M., H.I., Y.H., M.W.R., W.M.C.S., K.T., Y.O.; Methodology: Y.M., H.I., Y.H., M.W.R., K.T., Y.O.; Project Administration: K.T.,Y.O.; Validation, Y.H., M.W.R., W.M.C.S., K.T., Y.O.; Visualization: Y.M., K. T.; Supervision: K.T.,Y.O.; Writing – original draft: Y.M.; Writing – review & editing: K.T., Y.O. with participation of all authors.

**Competing interests:** Authors declare that they have no competing interests.

**Data and materials availability:** X-ray data are available free of charge from the Cambridge Crystallographic Data Centre under reference numbers CCDC 2586720 (**4**) and 2586721 (**5**). All other experimental, spectroscopic, crystallographic, and computational data are included in the supplementary materials.

**Additional information**

**Supplementary information** is available at XXXX.

**Correspondence and requests for materials** should be addressed K.T. or Y.O.

## Supplementary Files

This is a list of supplementary files associated with this preprint. Click to download.

- checkCIF4.pdf
- checkCIF5.pdf
- SI.pdf
